# Characteristics, Treatment, and Mortality of Patients Hospitalized for First ST-Segment Elevation Myocardial Infarction without Standard Modifiable Risk Factors in China

**DOI:** 10.31083/j.rcm2409249

**Published:** 2023-09-05

**Authors:** Weihong Guo, Yunfeng Wang, Aoxi Tian, Jiayi Yi, Jiamin Liu, Haibo Zhang, Jing Li, Shengshou Hu, Xi Li, Xin Zheng

**Affiliations:** ^1^National Clinical Research Center for Cardiovascular Diseases, State Key Laboratory of Cardiovascular Disease, Fuwai Hospital, National Center for Cardiovascular Diseases, Chinese Academy of Medical Sciences and Peking Union Medical College, 100037 Beijing, China; ^2^Central China Sub-center of the National Center for Cardiovascular Diseases, 450000 Zhengzhou, Henan, China; ^3^National Clinical Research Center for Cardiovascular Diseases, Shenzhen, Coronary Artery Disease Center, Fuwai Hospital Chinese Academy of Medical Sciences, 518057 Shenzhen, Guangdong, China

**Keywords:** ST-segment elevation myocardial infarction, risk factors, treatment, mortality

## Abstract

**Background:**

Little is known of the characteristics, treatment, and outcomes of patients with ST-segment elevation myocardial infarction (STEMI) but without standard modifiable cardiovascular risk factors (SMuRFs, including smoking, hypercholesterolemia, diabetes, and hypertension) in developing countries like China. Moreover, contributors to the excess mortality of such SMuRF-less patients remain unclear.

**Methods:**

This study was based on a nationally representative sample of patients presenting with STEMI and admitted to 162 hospitals in 31 provinces across mainland China between 2001 and 2015. We compared clinical characteristics, treatments, and mortality during hospitalization between patients with and without SMuRFs. We also investigated the possible causes of differences in mortality and quantified the contributors to excess mortality.

**Results:**

Among 16,541 patients (aged 65 ± 13 years; 30.0% women), 19.9% were SMuRF-less. These patients were older (69 vs. 65 years), experienced more cardiogenic shock and lower blood pressure at admission, and were less likely to be admitted to the cardiac ward compared to patients with SMuRFs. Moreover, SMuRF-less patients received treatment less often, including primary percutaneous coronary intervention (17.3% vs. 28.8%, *p* < 0.001), dual antiplatelet therapy (59.4% vs. 77.0%, *p* < 0.001), angiotensin-converting enzyme inhibitors/angiotensin receptor blockers (49.9% vs. 68.1%, *p* < 0.001), and statins (69.9% vs. 85.1%, *p* < 0.001). They had higher in-hospital mortality (18.5% vs. 10.5%, *p* < 0.001), with 56.1% of deaths occurring within 24 hours of admission. Although the difference in mortality decreased after adjusting for patient characteristics, it remained significant and concerning (odds ratio (OR) 1.41; 95% confidence interval (CI) 1.25–1.59). Mediation analysis found that, in patients without SMuRFs, underutilization of angiotensin-converting enzyme inhibitors/angiotensin receptor blockers and statins contributed to an excess mortality risk of 22.4% and 32.5%, respectively.

**Conclusions:**

Attention and action are urgently needed for STEMI patients without SMuRFs, given their high incidence and excess in-hospital mortality. The use of timely and adequate evidence-based treatments should be strengthened.

## 1. Introduction

Approximately 11–26% of patients hospitalized for ST-segment elevation myocardial infarction (STEMI) worldwide were found to have no standard modifiable cardiovascular risk factors (SMuRFs, including smoking, hypercholesterolemia, diabetes, and hypertension) at their first presentation [1, 2, 3, 4, 5, 6, 7]. Such patients are referred to as SMuRF-less, and their proportion among STEMI patients has been reported as 11.8% in China [2], 14.9% in Sweden [1], 19.2% in Australia [3], and 11.0–26.2% in the United States [4, 5, 6]. In some countries, this proportion has increased over time [3, 8]. Although they are commonly considered as low-risk populations and are often overlooked in research [1, 9], recent studies have reported that SMuRF-less patients experienced unexpectedly worse crude in-hospital mortality compared to those with SMuRFs [1, 2, 3, 5, 10, 11].

Comparisons regarding the management and outcomes between patients with and without SMuRFs are mainly from developed countries, while little is known in developing countries about the management of these patients in clinical practice. Furthermore, the reasons for the worse outcomes of SMuRF-less patients are still unclear, and observations were conflicting regarding the possible contributors. Some studies have suggested that the higher risk of mortality in SMuRF-less individuals can be fully accounted for by patient characteristics [10, 12], while others have indicated the underuse of treatments [1] and/or heterogeneity in patient characteristics [2]. Quantitative assessment of the major contributors should help determine the optimal measures for reducing the mortality of SMuRF-less patients.

Accordingly, we used the data from the China Patient-centered Evaluative Assessment of Cardiac Events Retrospective Study of acute myocardial infarction (AMI), namely China PEACE-Retrospective AMI Study [10]. This offers a nationally representative sample of patients who were hospitalized for STEMI in 162 hospitals across mainland China between 2001 and 2015. The aim of the present work was to compare the characteristics, therapies, and outcomes during hospitalization between STEMI patients with and without SMuRFs, and to explore the possible contributors to the differences in mortality.

## 2. Materials and Methods

### 2.1 Data Sources and Study Design

The China PEACE-Retrospective AMI Study protocol has been published earlier [13, 14]. Briefly, a two-stage random sampling procedure was used to draw nationally representative cases hospitalized for acute myocardial infarction (AMI) in 2001, 2006, and 2011. In the first stage, a simple random sampling process was used to include representative hospitals from five economic-geographic strata: eastern-rural, central-rural, western-rural, eastern-urban, and central/western-urban regions. These strata were used because socioeconomic levels and healthcare resources vary across categories (**Supplementary Fig. 1**). In the second stage, AMI patients from the sampled hospitals were drawn using systematic random sampling methods. AMI hospitalizations were identified by International Classification of Diseases (ICD) codes, Ninth Revision (410.xx) and Tenth Revision (I21.xx), if available, or by the principal discharge diagnosis. This study also included patients admitted in 2015 using the same method.

Trained staff retrieved data from medical charts using clear abstraction approaches and standardized data definitions. Each medical record was copied by the participating hospital and transmitted to the central abstraction center. All abstractors received central training for two weeks. Those who could extract 10 sample medical records with more than 98% accuracy after training received certification. Rigorous monitoring was employed at each stage to ensure the accuracy of abstraction. Data quality was monitored by randomly auditing 5% of the abstracted records. The overall accuracy exceeded 98% [13, 14].

The central ethics committee of the Chinese National Center for Cardiovascular Diseases approved this study. Given the retrospective nature, written patient consent was not required. Ethics approval was also obtained from all participating hospitals.

### 2.2 Study Population

Patients with a discharge diagnosis of STEMI were included, which was determined by combining the diagnosis at discharge with the results of electrocardiograms (ECGs). In cases where there was no definitive diagnosis from the local hospital, cardiologists from the coordinating center reviewed the medical logs and ECGs to determine the STEMI diagnosis. An independent cardiologist who did not take part in abstraction validated the AMI type by reviewing ECGs in randomly selected records. The concordance rate for the selected cases was 94.7%.

Patients were excluded if they had an established myocardial infarction, percutaneous coronary intervention (PCI), or coronary artery bypass graft before admission, or if their STEMI occurred during hospitalization. Also excluded were patients with missing baseline data on SMuRFs, patients transferred in or out because their hospitalization was truncated, and patients discharged alive within the first 24 hours, given that they were very likely to have left against medical advice and had very little time to receive treatment (**Supplementary Fig. 2**).

### 2.3 Definition of Variables

Similar to a previous study [1], we defined SMuRFs as having at least one of the following modifiable risk factors: current smoking, hypercholesterolemia, diabetes, or hypertension. The definition of current smoking is having smoked regularly (at least one cigarette per day) during the last six months. Hypercholesterolemia was defined as having a low-density lipoprotein cholesterol concentration ≥3.4 mmol/L, or a total cholesterol concentration ≥5.2 mmol/L during the index admission, or an established or new diagnosis of hypercholesterolemia. Diabetes was defined as having an established or new diagnosis of diabetes, and hypertension was defined as having an established or new diagnosis of hypertension. Blood pressure in the acute phase and fasting glucose were excluded from the definitions, as both are influenced by the neurohormonal response to AMI, which was consistent with the previous study [1].

The use of in-hospital therapies recommended by the guidelines for the management of STEMI was assessed, which included aspirin, ${\mathrm{P2Y}}_{12}$ inhibitors, dual antiplatelet therapy (DAPT), β-blockers, angiotensin-converting enzyme (ACE) inhibitors/angiotensin receptor blockers (ARBs), statins, and reperfusion therapy (primary PCI or fibrinolytic therapy) [15]. We assessed the usage rates of therapy merely in the eligible patients (i.e., those with no documented contraindications) after excluding those who died within the first 24 hours, since these may not have sufficient opportunity to be treated (**Supplementary Method 1**). To assess PCI and diagnostic catheterization, the study population was limited to the patients admitted to the hospitals with PCI capacity.

In-hospital mortality was defined as death or treatment withdrawal due to a terminal condition. The Chinese Government uses death or withdrawal of treatment as an indicator of hospital quality [16]. At the coordinating hospitals, cardiologists judged the clinical status of patients withdrawn from treatment based on their medical records. Composite complications included death, treatment withdrawal due to a terminal condition, congestive heart failure, re-infarction, ischemic stroke, or cardiogenic shock.

### 2.4 Statistical Analysis

Continuous variables were analyzed using *t*-tests or non-parametric equivalent tests and presented as medians with interquartile ranges. Categorical variables were listed as percentages, and analysis was performed using χ^2^ tests.

To explore the link between SMuRF-less status and therapies received, mixed models with hospitals as a random intercept were used to account for age, sex, medical histories, clinical profiles at admission, and admission ward.

Survival curves and hazard functions for in-hospital mortality were plotted. Mixed effect models were also used to assess whether SMuRF-less was independently associated with mortality, accounting for all explanatory variables stepwise. The models used in the study were: an unadjusted model; model 1 (adjusted for age and sex); model 2 (adjusted for model 1, medical history [stroke, atrial fibrillation, chronic renal disease, heart failure, peripheral arterial disease], and clinical characteristics at admission [systolic blood pressure [SBP], heart rate, chest discomfort, cardiac arrest, cardiogenic shock, stroke]); and model 3 (adjusted for model 2, pharmacotherapies during hospitalization [aspirin, ${\mathrm{P2Y}}_{12}$ inhibitors, DAPT, β-blockers, statins, ACE inhibitors/ARBs], as well as reperfusion therapy [primary PCI and fibrinolysis]). Stratified analyses were performed according to sex. Given that there were small differences in length of hospital stay between patients with and without SMuRFs, we also compared the adjusted 7-day mortality as a sensitivity analysis.

To explore the possible contribution of each treatment to the disparities in mortality, the effect of each treatment was investigated using age- and sex-adjusted analyses, taking the SMuRF-less status into consideration. Formal mediation analyses were also performed to examine the extent to which specific variables (including all clinical profiles and treatments) might contribute to in-hospital mortality in SMuRF-less patients. A mediator was defined as a variable that lies along the causal chain connecting the predictor and mortality. Traditionally, mediators are often adjusted in the assessment of causal association. Meaningful associations between mortality and the predictor could thus be removed, leading to incorrect conclusions of no association. Therefore, formal mediation analyses would facilitate identifying potential factors to explain the higher mortality among SMuRF-less and SMuRF patients. We calculated the percent mediation by dividing the indirect effect with the total effect and presented the proportion of the total effect attributable to the mediator. These analyses were performed with the mma package, as detailed elsewhere [17].

The present study did not impute missing values for SBP and heart rate in the models, since the missing data was minimal (<0.2% of patients).

All statistical analyses were performed by SAS (version 9.4, SAS Institute Inc., Cary, NC, USA) and software R (version 4.2.1, R Foundation for Statistical Computing, Vienna, Austria). A two-sided *p* value < 0.05 was considered statistically significant.

## 3. Results

### 3.1 Patient Characteristics

We included 16,541 patients (4970 women [30.0%], median age 66 years [56–74]), of whom 3288 (19.9%) had no documented SMuRFs and were henceforth referred to as SMuRF-less.

Baseline characteristics are presented in Table 1. SMuRF-less individuals were older (69 years [59–76] vs. 65 years [55–74], *p* < 0.001) and more often female (33.6% vs. 29.2%, *p* < 0.001) compared to those with SMuRFs. The duration from the onset of symptoms to hospital admission did not differ between the two groups (15 h [4–72] vs. 16 h [4–72], *p* = 0.970). SMuRF-less individuals were less likely to have chest discomfort or to be admitted to the cardiac ward. Despite similar median troponin concentrations (37.7-fold vs. 37.5-fold the upper limit of normal), we observed a longer delay in measuring cardiac enzymes in SMuRF-less patients (107 min [12–630] vs. 93 min [7–340], *p* < 0.001). SMuRF-less patients also had a significantly lower SBP (120 mmHg [100–130] vs. 130 mmHg [111–150], *p* < 0.001) and a greater proportion of SBP <90 mmHg (9.7% vs. 4.4%, *p* < 0.001) and cardiogenic shock (9.4% vs. 6.1%, *p* < 0.001).

**Table 1.** **Baseline characteristics of SMuRF-less patients and of patients with at least one SMuRF**.

Variable			SMuRF-less (N = 3288)	≥1 SMuRF (N = 13,253)	*p *value
Age (years), median (interquartile range)			69 (59, 76)	65 (55, 74)	< 0.001
Age (years), N (%)					
	<40		79 (2.4)	372 (2.8)	< 0.001
	40–59		789 (24.0)	4345 (32.8)	
	60–79		1873 (57.0)	7158 (54.0)	
	≥80		547 (16.6)	1378 (10.4)	
Female, N (%)			1104 (33.6)	3866 (29.2)	< 0.001
Medical history, N (%)					
	Stroke		251 (7.6)	1705 (12.9)	< 0.001
	Peripheral arterial disease		1 (0.0)	16 (0.1)	0.224
	Atrial fibrillation		29 (0.9)	105 (0.8)	0.607
	Chronic renal disease		37 (1.1)	300 (2.3)	< 0.001
	Heart failure		28 (0.9)	79 (0.6)	0.102
Time from symptom onset to admission (hours), median (interquartile range)			15 (4, 72)	16 (4, 72)	0.970
Clinical profile at admission					
	Chest discomfort, N (%)		2940 (89.4)	12,265 (92.5)	< 0.001
	Cardiogenic shock, N (%)		310 (9.4)	803 (6.1)	< 0.001
	Acute stroke, N (%)		56 (1.7)	245 (1.8)	0.576
	Cardiac arrest, N (%)		52 (1.6)	168 (1.3)	0.160
	SBP (mmHg), median (interquartile range)		120 (100, 130)	130 (111, 150)	< 0.001
	SBP (mmHg), N (%)				
		<90	317 (9.7)	581 (4.4)	< 0.001
		90–139	2365 (72.1)	7652 (57.8)	
		≥140	598 (18.2)	4997 (37.8)	
	Heart rate (beats/min), median (interquartile range)		76 (64, 90)	78 (66, 90)	0.139
	Left ventricular ejection fraction ${\mathrm{(\%)}}^{a}$		55 (46, 61)	56 (48, 62)	0.003
	CRP ${\mathrm{(mg/L)}}^{a}$		9.4 (4.1, 35.9)	7.0 (3.0, 28.1)	0.106
	Total cholesterol ${\mathrm{(mmol/L)}}^{a}$		4.0 (3.5, 4.5)	4.7 (3.9, 5.4)	< 0.001
	LDL-C ${\mathrm{(mmol/L)}}^{a}$		2.3 (1.9, 2.8)	2.8 (2.2, 3.5)	< 0.001
	HDL-C ${\mathrm{(mmol/L)}}^{a}$		1.1 (0.9, 1.3)	1.1 (0.9, 1.3)	0.859
	Triglycerides ${\mathrm{(mmol/L)}}^{a}$		1.1 (0.8, 1.5)	1.3 (1.0, 2.0)	< 0.001
	Glucose ${\mathrm{(mmol/L)}}^{a}$		6.4 (5.4, 8.2)	7.0 (5.7, 9.5)	< 0.001
	Hemoglobin ${\mathrm{(g/L)}}^{a}$		130 (117, 143)	135 (122, 148)	< 0.001
	Hematocrit ${\mathrm{(\%)}}^{a}$		39 (35, 42)	40 (36, 44)	< 0.001
	EGFR ${\mathrm{(mL/min per 1.73 m²)}}^{a}$		80.5 (59.9, 102.0)	84.2 (64.4, 105.8)	< 0.001
	Duration from admission to cardiac enzyme measurement ${\mathrm{(minutes)}}^{a}$		107 (12, 630)	93 (7, 340)	< 0.001
	Concentration of troponin ${\mathrm{(multiple of upper limit of normal)}}^{a}$		37.7 (5.2, 146.0)	37.5 (6.1, 168.7)	0.142
Admission ward, N (%)					
	Cardiac ward		1375 (41.8)	7263 (54.8)	< 0.001
	Non-Cardiac ward		1913 (58.2)	5990 (45.2)	< 0.001
Hospital characteristics, N (%)					
	Teaching hospital		2267 (68.9)	10,633 (80.2)	< 0.001
	PCI-capable hospital		1820 (55.4)	9595 (72.4)	< 0.001
	Hospital with CCU		1031 (31.4)	2676 (20.2)	< 0.001
Economic-geographic region, N (%)					
	Central		942 (28.6)	2992 (22.6)	< 0.001
	Eastern		1620 (49.3)	7578 (57.2)	
	Western		726 (22.1)	2683 (20.2)	
Urban/rural, N (%)					
	Urban		1672 (50.9)	8421 (63.5)	< 0.001
	Rural		1616 (49.1)	4832 (36.5)	< 0.001

Abbreviations: SBP, systolic blood pressure; CRP, C-reactive protein; LDL-C, low-density lipoprotein cholesterol; HDL-C, high-density lipoprotein cholesterol; EGFR, estimated glomerular filtration rate; PCI, percutaneous coronary intervention; CCU, cardiac care unit; SMuRF, standard modifiable cardiovascular risk factor; N, number. ^a^ Among patients with available data.

### 3.2 In-Hospital Treatment

The proportion of eligible patients for aspirin, ${\mathrm{P2Y}}_{12}$ inhibitors, DAPT, and statins was not different between patients with and without SMuRFs after excluding those who stayed in hospital for ≤24 hours. However, the SMuRF-less group was less likely to be eligible for β-blockers (74.6% vs. 80.3%, *p* < 0.001), ACE inhibitors/ARBs (93.5% vs. 96.2%, *p* < 0.001) and reperfusion therapy (51.5% vs. 54.5%, *p *= 0.004) (**Supplementary Table 1**).

Among eligible patients, those in the SMuRF-less group were less likely to be treated with medications, including aspirin (89.0% vs. 94.7%, *p* < 0.001), ${\mathrm{P2Y}}_{12}$ inhibitors (61.1% vs. 78.6%, *p* < 0.001), DAPT (59.4% vs. 77.0%, *p* < 0.001), β-blockers (78.3% vs. 85.7%, *p* < 0.001), ACE inhibitors/ARBs (49.9% vs. 68.1%, *p* < 0.001), and statins (69.9% vs. 85.1%, *p* < 0.001) (Table 2). Additionally, the SMuRF-less group had lower utilization of primary PCI (17.3% vs. 28.8%, *p* < 0.001), but similar use of fibrinolytic therapy (35.0% vs. 32.9%, *p *= 0.100). These differences persisted after adjusting for age, sex, medical history, clinical characteristics at admission, and admission ward. The most marked differences were observed for the use of ACE inhibitors/ARBs (odds ratio [OR] 0.56; 95% confidence interval [CI] 0.51–0.61) and statins (OR 0.61; 95% CI 0.53–0.70) (Table 2).

**Table 2.** **In-hospital therapies and procedures amongst eligible patients**.

		SMuRF-less	≥1 SMuRF	Adjusted OR (SMuRF-less versus ≥1 ${\mathrm{SMuRF)}}^{a}$	*p* value
Medical therapies, N (%)					
	Aspirin within 24 h	2452 (83.7)	11,239 (88.9)	0.77 (0.68, 0.88)	< 0.001
	${\mathrm{P2Y}}_{12}$ inhibitor within 24 h	1606 (55.0)	9051 (71.8)	0.72 (0.64, 0.81)	< 0.001
	DAPT within 24 h	1561 (53.5)	8757 (69.6)	0.76 (0.67, 0.85)	< 0.001
	β-blocker within 24 h	821 (37.4)	4566 (44.7)	0.92 (0.82, 1.02)	0.117
	ACE inhibitor/ARB within 24 h	1384 (50.2)	8231 (67.3)	0.57 (0.52, 0.62)	< 0.001
	Statin within 24 h	1901 (64.5)	9922 (78.1)	0.76 (0.67, 0.85)	< 0.001
	Aspirin	2606 (89.0)	11,963 (94.7)	0.64 (0.54, 0.76)	< 0.001
	${\mathrm{P2Y}}_{12}$ inhibitor	1784 (61.1)	9908 (78.6)	0.67 (0.58, 0.76)	< 0.001
	DAPT	1734 (59.4)	9687 (77.0)	0.67 (0.59, 0.76)	< 0.001
	β-blocker	1721 (78.3)	8745 (85.7)	0.66 (0.58, 0.75)	< 0.001
	ACE inhibitor or ARB	1376 (49.9)	8333 (68.1)	0.56 (0.51, 0.61)	< 0.001
	Statin	2059 (69.9)	10,814 (85.1)	0.61 (0.53, 0.70)	< 0.001
Procedures, N (%)					
	Cardiac catheterization	715 (43.9)	4841 (52.4)	0.88 (0.77, 1.01)	0.070
Coronary artery lesion, ≥50% stenosis, N ${\mathrm{(\%)}}^{b}$					
	Intermediate	5 (1.4)	55 (2.2)	/	0.547
	Left anterior descending artery	295 (80.4)	2104 (84.8)	/	0.032
	Left circumflex artery	162 (44.1)	1426 (57.5)	/	< 0.001
	Right coronary artery	246 (67.1)	1697 (68.4)	/	0.791
	Left main coronary artery	25 (6.8)	137 (5.5)	/	0.399
	Multivessel disease, ≥50% stenosis, N ${\mathrm{(\%)}}^{b}$	236 (64.3)	1800 (72.6)	/	0.001
	Non-obstructive coronary disease, N ${\mathrm{(\%)}}^{b}$	14 (3.8)	51 (2.1)	/	0.035
	PCI (non-primary)	322 (19.8)	2152 (23.3)	0.86 (0.75, 0.99)	0.038
	CABG	6 (0.2)	48 (0.4)	0.83 (0.35, 2.00)	0.684
Reperfusion therapies, N (%)					
	Primary PCI	262 (17.3)	1992 (28.8)	0.80 (0.66, 0.98)	0.028
	Fibrinolytic therapy	532 (35.0)	2275 (32.9)	0.88 (0.76, 1.01)	0.065

Abbreviations: DAPT, dual antiplatelet therapy; ACE, angiotensin-converting enzyme; ARB, angiotensin receptor blocker; PCI, percutaneous coronary intervention; CABG, coronary artery bypass grafting; SMuRF, standard modifiable cardiovascular risk factor; OR, odds ratio; N, number. ^a^ Adjusted for age, sex, admission ward, medical history (previous stroke, previous atrial fibrillation, previous chronic renal disease, previous heart failure, previous peripheral arterial disease), and clinical characteristics at admission (chest discomfort, cardiogenic shock, stroke, cardiac arrest, heart rate, and systolic blood pressure). ^b^ Data were only available in patients who underwent coronary angiography in 2015.

### 3.3 In-Hospital Outcomes

**Fig. 1.**
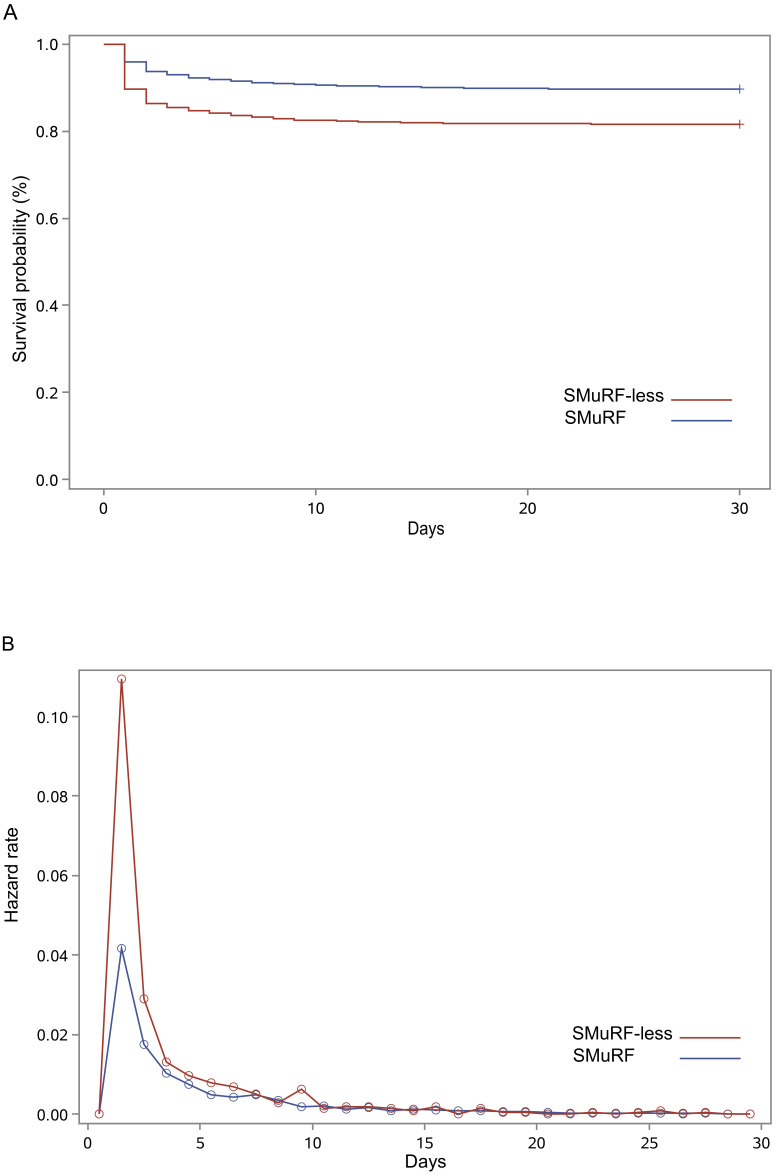
**Survival curves and hazard function curves based on SMuRF status**. (A) Kaplan-Meier survival curves for in-hospital mortality until discharge. (B) Hazard function for mortality during hospitalization. SMuRF, standard modifiable cardiovascular risk factor.

Individuals without SMuRFs experienced significantly higher crude in-hospital mortality (18.5% vs. 10.5%, *p* < 0.001) and composite complications (26.0% vs. 19.0%, *p* < 0.001) (**Supplementary Table 2**). 56.1% of deaths occurred during the first 24 hours in SMuRF-less patients compared to 38.9% in SMuRF patients. The 24-hour mortality rate was more than 2-fold higher in the SMuRF-less group than in patients with at least one SMuRF (10.4% vs. 4.1%, *p* < 0.001). For patients who survived the first 24 hours of admission, the disparity in mortality between the two groups narrowed but remained significant (9.1% vs. 6.7%, *p* < 0.001) (**Supplementary Table 2**). The length of hospital stay was shorter in SMuRF-less patients (9 days [IQR 5–14] vs. 11 days [7–15], *p* < 0.001). Fig. 1A shows the survival curves for the two patient groups. In both, the highest hazard function (instantaneous risk) for death was within the first 24 hours (Fig. 1B). SMuRF-less individuals had a consistently higher risk of in-hospital mortality in all subgroups examined (**Supplementary Table 3**).

**Fig. 2.**
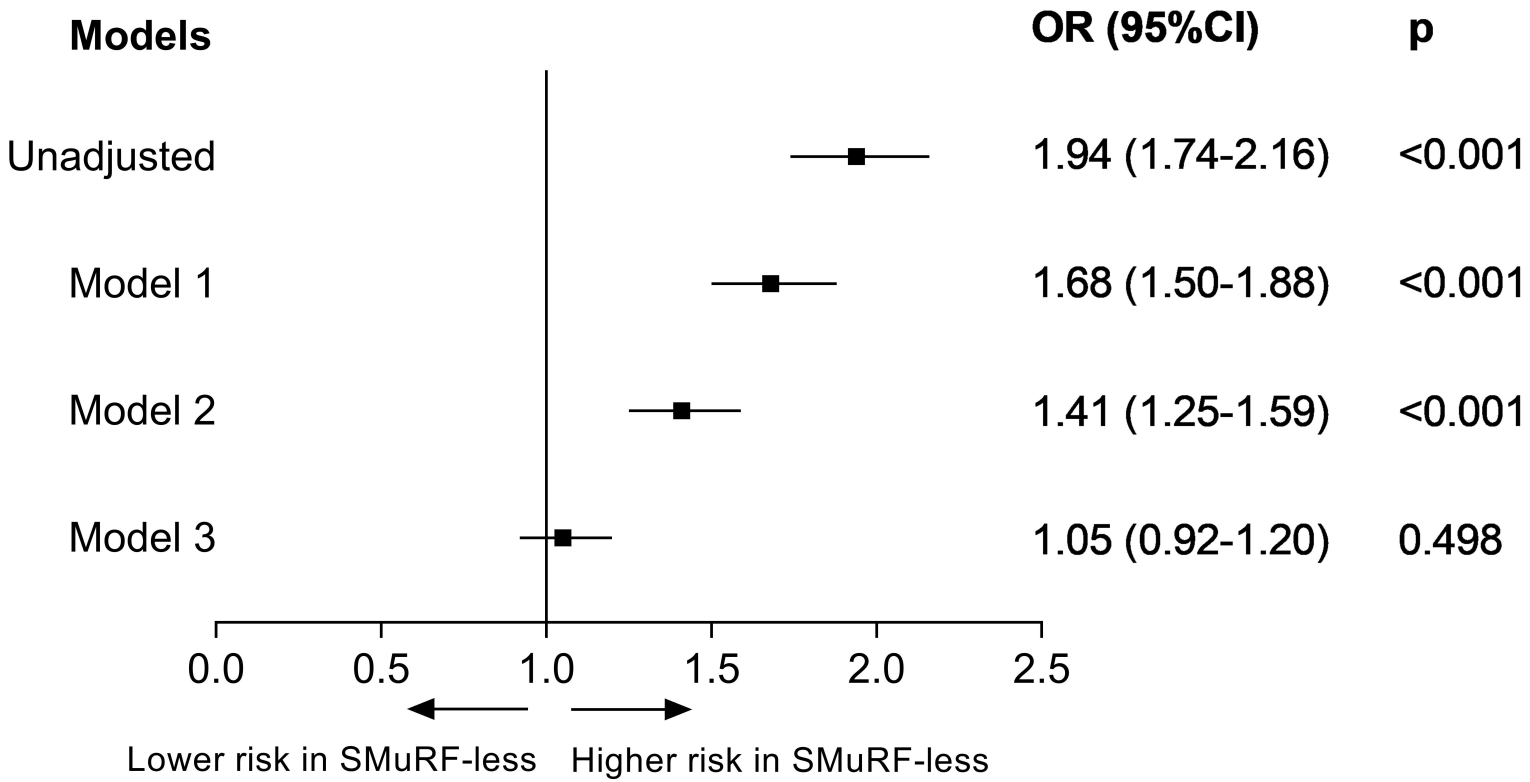
**Adjusted odds ratio for mortality during hospitalization between patients with and without SMuRFs**. Model 1: adjusted for age and sex; Model 2: adjusted for the variables in model 1 and for the clinical profiles (including previous stroke, previous atrial fibrillation, previous chronic renal disease, previous heart failure, previous peripheral arterial disease, chest discomfort, cardiac arrest at admission, cardiogenic shock at admission, stroke at admission, heart rate, and systolic blood pressure); Model 3: adjusted for the variables in model 2 and for in-hospital pharmacotherapies (including aspirin, ${\mathrm{P2Y}}_{12}$ inhibitor, dual antiplatelet therapy, β-blocker, angiotensin-converting enzyme inhibitor/angiotensin receptor blocker, statin) and reperfusion therapy (fibrinolytic therapy, primary percutaneous coronary intervention). SMuRF, standard modifiable cardiovascular risk factor; OR, odds ratio.

After adjustment for age and sex, the SMuRF-less group had a 68% greater risk of in-hospital death (OR 1.68; 95% CI 1.50–1.88) (Fig. 2). This difference was reduced after adjusting for clinical profiles (OR 1.41; 95% CI 1.25–1.59). After further adjustment for in-hospital treatment, the difference was no longer significant (OR 1.05; 95% CI 0.92–1.20). Among all of the individual treatments, the use of ACE inhibitors/ARBs (OR 1.29; 95% CI 1.15–1.45) or statins (OR 1.36; 95% CI 1.21–1.54) resulted in the largest reduction in the OR of mortality for SMuRF-less patients (**Supplementary Fig. 3**). The results of these analyses agreed with those for sex stratification (**Supplementary Figs. 4,5**) and for the use of a 7-day timeframe (**Supplementary Figs. 6**,**7**). However, after excluding individuals who died within 24 hours of admission and after adjusting for age and sex, the disparity in in-hospital mortality was only marginally significant (OR 1.16; 95% CI 1.00–1.35) (**Supplementary Fig. 8**).

### 3.4 Mediators of Excess Mortality

Table 3 lists the mediating factors and their percent mediation in the overall population. Mediating factors were estimated to account for 92.5% of the excess in-hospital mortality observed in SMuRF-less patients compared to those who had SMuRFs. Although 23.0% of the excess mortality in SMuRF-less patients was mediated by worse clinical profiles, the majority (69.1%) was mediated by suboptimal in-hospital treatment. The contributions from in-hospital statin and ACE inhibitor/ARB treatments were 32.5% and 22.4%, respectively, accounting for the largest proportion of difference in mortality. The mediation analyses were repeated in the eligible patients, with similar results obtained. Among the eligible patients for ACE inhibitors/ARBs, 23.4% of the excess in-hospital mortality was due to the underuse of this treatment, while among the eligible ones for statins, 30.6% of excess mortality was due to treatment underuse.

**Table 3.** **Mediation analysis for excess in-hospital mortality in SMuRF-less patients (overall population)**.

			Mediated ${\mathrm{effect}}^{a}$
Total indirect ${\mathrm{effect}}^{b}$			92.5%
Indirect effect through:			
	Clinical profile		23.0%
		Systolic blood pressure	9.6%
		Cardiogenic shock at admission	3.7%
		Acute stroke at admission	1.9%
	In-hospital treatment		69.1%
		In-hospital statin	32.5%
		In-hospital ACE inhibitor/ARB	22.4%
		In-hospital β-blocker	7.7%
		Primary PCI	7.4%
		In-hospital aspirin	3.4%
		In-hospital ${\mathrm{P2Y}}_{12}$ inhibitor	2.4%
		In-hospital DAPT	1.6%

Abbreviations: ACE, angiotensin-converting enzyme; ARB, angiotensin receptor blocker; PCI, percutaneous coronary intervention; DAPT, dual antiplatelet therapy; SMuRF, standard modifiable cardiovascular risk factor. ^a^ Percent contribution from each mediator to the excess in-hospital mortality of the SMuRF-less group compared with SMuRF group. ^b^ Due to correlation and overlapping mediation effects among mediators (reflected in the total indirect impact but not in the individual mediators), the sum of the effect for individual mediators may not equal the total indirect effect.

After excluding patients who died within 24 hours of hospitalization, mediation analyses produced similar results to those of the overall population, i.e., 30.3% of the difference in mortality between SMuRF-less and SMuRF patients could be explained by the underuse of ACE inhibitors, and 13.8% by the underuse of statins.

Given that the most prominent in-hospital mortality difference was within the first 24 hours of hospitalization, we performed a sensitivity analysis using mediation analysis to examine the potential contributors to excess mortality in SMuRF-less patients within this period (**Supplementary Table 4**). The clinical profiles and treatments within 24 hours jointly accounted for 57.9% of the relationship between the SMuRF-less status and mortality within 24 hours. The underuse of ${\mathrm{P2Y}}_{12}$ inhibitor therapy within 24 hours accounted for the largest proportion (13.0%) (**Supplementary Table 4**).

## 4. Discussion

Almost one in five patients hospitalized for STEMI in China has no SMuRFs. Compared to patients with SMuRFs, SMuRF-less individuals presented with a more serious condition and received fewer evidence-based therapies, even among the eligible patients. The higher risk of in-hospital mortality in SMuRF-less patients was largely explained by the differences in the severity of illness and in-hospital treatments. In particular, the underuse of statins and ACE inhibitors/ARBs contributed to most of the excess risk of death in SMuRF-less patients.

In this nationally representative sample of Chinese patients hospitalized for STEMI, the proportion of SMuRF-less patients was similar to that reported in developed countries [1, 3, 12]. However, it was almost 2-fold higher than reported in the CAMI (China Acute Myocardial Infarction) registry study [2], possibly due to the differences in study design. The CAMI registry study prospectively enrolled patients from a non-random sample, therefore potentially missing some patients who died during the very early phase of hospitalization [2]. Of note, we observed that >50% of the in-hospital mortality in SMuRF-less patients took place during the initial 24 hours of admission. Our study retrospectively included patients through a random sampling procedure, thus reflecting the actual proportion of this patient population in China.

In line with previous studies, SMuRF-less patients were sicker and older [1, 2, 10, 18, 19]. The older age may increase the absolute baseline risk of AMI, independent of SMuRFs [11, 20]. In addition, SMuRF-less patients presented more often with cardiogenic shock at admission, and had higher mortality within the first 24 hours. Reduced or absent myocardial ischemic preconditioning, or differences in plaque composition, may partially explain the more severe presentation of SMuRF-less patients [7, 21]. Recent studies also reported a larger infarct size, worse flow (grade 0/1), and less calcification in SMuRF-less patients [7, 22].

As reported earlier, SMuRF-less patients received fewer evidence-based therapies [2, 3, 10, 11, 23]. Potential explanations for this undertreatment include: (1) less eligibility for therapy due to a more severe condition, (2) limited treatment opportunities due to early death, (3) delayed diagnosis due to atypical symptoms, and (4) treatment bias because of lower risk factors. Here, we extended previous studies by only focusing on patients who had no contraindications and survived the first 24 hours upon admission. Nevertheless, we found this group remained undertreated. Importantly, we observed that fewer SMuRF-less patients had chest discomfort and were admitted to the cardiac ward, possibly reflecting early diagnostic uncertainty [24]. The delayed diagnosis may lead to a delay in the initial management and, subsequently, to undertreatment. Reperfusion therapy, in particular, is required to be performed within a recommended time window [25]. The lack of hypertension and hypercholesterolemia in individuals without SMuRFs may also partially explain their undertreatment [12, 22, 26]. These findings suggest that there might be an unreasonable risk factor-driven treatment bias, i.e., only patients with risk factors would be treated with ACE inhibitors/ARBs or statins in clinical practice.

A higher rate of in-hospital mortality was observed in SMuRF-less patients. Particularly, the most prominent excess mortality occurred within the first 24 hours. This finding extended previous studies [1, 3, 5, 11, 12], and first restricted the difference in outcome to the very early stage. The worse baseline profiles and suboptimal treatment of SMuRF-less patients contribute to the excess in-hospital mortality. Mediation analyses allowed us to better identify the contributors, of which the underuse of clinical care was observed as the most important contributor. In particular, the suboptimal use of statins and ACE inhibitors/ARBs contributed the largest proportion to the excess risk of in-hospital mortality. And the immediate underuse of antiplatelet therapy was the largest contributor to the excess risk of death within 24 hours. Our results concur with prior research showing that immediate initiation of statins [27, 28], ACE inhibitors [29, 30], and antiplatelet therapy reduce in-hospital mortality after STEMI [31]. Despite the benefits of early reperfusion therapy, its impact on mortality between the two groups was modest in this study. As mentioned above, the underuse of statins and ACE inhibitors/ARBs might be due to risk-driven bias, while the underuse of antiplatelet therapy could be due to the delay in diagnosis.

As the first nationally representative study to describe the characteristics of STEMI patients without SMuRFs in developing countries, our study has several clinical implications. First, physicians should be aware of the disparities in presentation between SMuRF-less and SMuRF patients to minimize the delays in recognition and triage. Second, our mediation analyses first found that the underuse of ACEI/ARB and statins explained most of the excess in-hospital mortality of SMuRF-less patients,emphasizing the importance of equitable treatments for this population. It is also worth highlighting that suboptimal treatments exist not only in SMuRF-less patients but also in SMuRF patients, suggesting that there is room to improve overall care for all AMI patients. Prior studies also showed suboptimal prescriptions for secondary prevention and poor risk factor control in patients with risk factors [32, 33]. Quality improvement programs and the establishment of national systemic measures of performance may provide additional impetus to improve the care with AMI [34]. Additionally, during primary care and specialist follow-ups, the importance of medication adherence should be emphasized at each consultation, and referral for additional support should be recommended if necessary. Third, the large number of SMuRF-less patients indicates the need to explore new markers for early atherosclerosis and improve the available risk tools in order to prevent AMI events, as traditional risk assessment methods are inadequate. Large-scale genome-wide association studies have found 55 genetic loci linked to coronary artery disease, with 66% of these loci being unrelated to conventional cardiovascular risk factors [35]. Imaging and biochemistry studies have also detected subclinical atherogenesis, even in healthy SMuRF-less individuals [36, 37]. It is therefore important to develop better risk prediction tools, including genetic, metabolomic, inflammatory, and imaging markers. Fourth, the primary prevention strategy in SMuRF-less individuals should be reconsidered. The US Preventive Services Task Force recently advised that clinicians offer or refer to behavioral counseling interventions to encourage physical activity and healthy eating to prevent cardiovascular disease in people without traditional risk factors [38]. Fifth, about 40% of the excess mortality of SMuRF-less patients occurring within 24 hours of admission has no obvious explanation. This indicates there are knowledge gaps in the underlying biological mechanisms responsible for early death after the onset of STEMI. Our findings could enable a better understanding of this often overlooked population in developing countries, where data is still quite limited.

Our study has several limitations. First, the data were retrospectively collected based on medical records. The lack of quantified variables, such as socioeconomic factors, lifestyle, and lipoprotein (a), might cause some residual confounding. It has been demonstrated that low lipoprotein (a) concentration (<7 nmol/L) was also associated with an increased risk of death following AMI, and a part of this association was probably attributable to the excess risk of heart failure [39]. Second, some risk factors might have been missed, since the approaches to risk factor diagnosis may vary slightly in different hospitals. To minimize the misclassification of SMuRF status, we included medical history, laboratory measurements, and new diagnoses during hospitalization to define SMuRFs. Third, some patients might have been too ill to accurately recall their medical history or report their risk factors, thereby resulting in misclassification. Extensive analyses were conducted to address this concern. In each case, the mortality in SMuRF-less patients was consistently higher, regardless of whether we examined in-hospital mortality according to age group or clinical severity (cardiac shock at admission), or whether patients who died within 24 hours of admission were excluded. Furthermore, it is uncommon not to obtain any history either from previous records or family members’ interviews in clinical practice [11]. Fourth, the most recent data for this study was from 2015. Nevertheless, the higher mortality observed for SMuRF-less STEMI patients is still concerning and requires more efforts to close the gaps.

## 5. Conclusions

Almost one-fifth of patients hospitalized for STEMI in China had no SMuRFs. These patients were more ill than those with SMuRFs, with half of them dying within 24 hours of hospitalization. Moreover, they received fewer recommended therapies and had higher hospital mortality rates, mainly due to suboptimal treatment. The underuse of ACE inhibitors/ARBs and statins explained a large percentage of the excess mortality of both overall and eligible SMuRF-less patients, which highlights the need to optimize evidence-based health care to address the disparity in outcome.

## Data Availability

The datasets used and/or analyzed during the current study are available from the corresponding author on reasonable request.

## Abbreviations

STEMI, ST-segment elevation myocardial infarction; SMuRFs, standard modifiable cardiovascular risk factors; China PEACE-Retrospective AMI, China Patient-centered Evaluative Assessment of Cardiac Events Retrospective Study of Acute Myocardial Infarction; AMI, acute myocardial infarction; ECGs, electrocardiograms; PCI, percutaneous coronary intervention; DAPT, dual antiplatelet therapy; ACE, angiotensin-converting enzyme; ARB, angiotensin receptor blocker; SBP, systolic blood pressure; OR, odds ratio; CI, confidence interval; CAMI, China Acute Myocardial Infarction.

## Supplementary Material

Supplementary material associated with this article can be found, in the online version, at https://doi.org/10.31083/j.rcm2409249.
