# Supplementary material for: Characteristics, Treatment, and Mortality of Patients Hospitalized for First ST-Segment Elevation Myocardial Infarction without Standard Modifiable Risk Factors in China
Source: Rev Cardiovasc Med. 2023 Sep 5;24(9):249. doi: 10.31083/j.rcm2409249 (PMC11270112; doi:10.31083/j.rcm2409249)
Supplement: Supplementary file 1 [file 2153-8174-24-9-249-s1.zip › 2153-8174-24-9-249-s1.docx]

Online-Only Supplements

Supplementary Fig. 1. Geographic distribution of participating hospitals in the China PEACE-Retrospective AMI study

Supplementary Fig. 2. Study flow chart

Supplementary Method 1. Eligible candidates for treatments

Supplementary Table 1. Proportion of patients eligible for treatment according to SMuRF status among patients who survived the first 24 hours of admission

Supplementary Table 2. In-hospital outcomes according to SMuRF status in the overall population

Supplementary Table 3. Subgroup analysis for crude in-hospital mortality in the overall population

Supplementary Fig. 3. Associations between SMuRF-less status and in-hospital mortality analyzed with mixed models in order to examine the potential effect of each treatment during hospitalization

Supplementary Fig. 4. Associations between SMuRF-less status and in-hospital mortality stratified by sex

Supplementary Fig. 5. Associations between SMuRF-less status and in-hospital mortality stratified by sex and analyzed with mixed models in order to examine the potential effect of each treatment during hospitalization

Supplementary Fig. 6. Associations between SMuRF-less status and 7-day mortality

Supplementary Fig. 7. Associations between SMuRF-less status and 7-day mortality analyzed with mixed models in order to examine the potential effect of each treatment during hospitalization

Supplementary Fig. 8. Associations between SMuRF-less status and in-hospital mortality for patients who survived the first 24 hours of admission

Supplementary Table 4. Mediation analysis of the associations between SMuRF-less status and mortality within 24 hours of admission


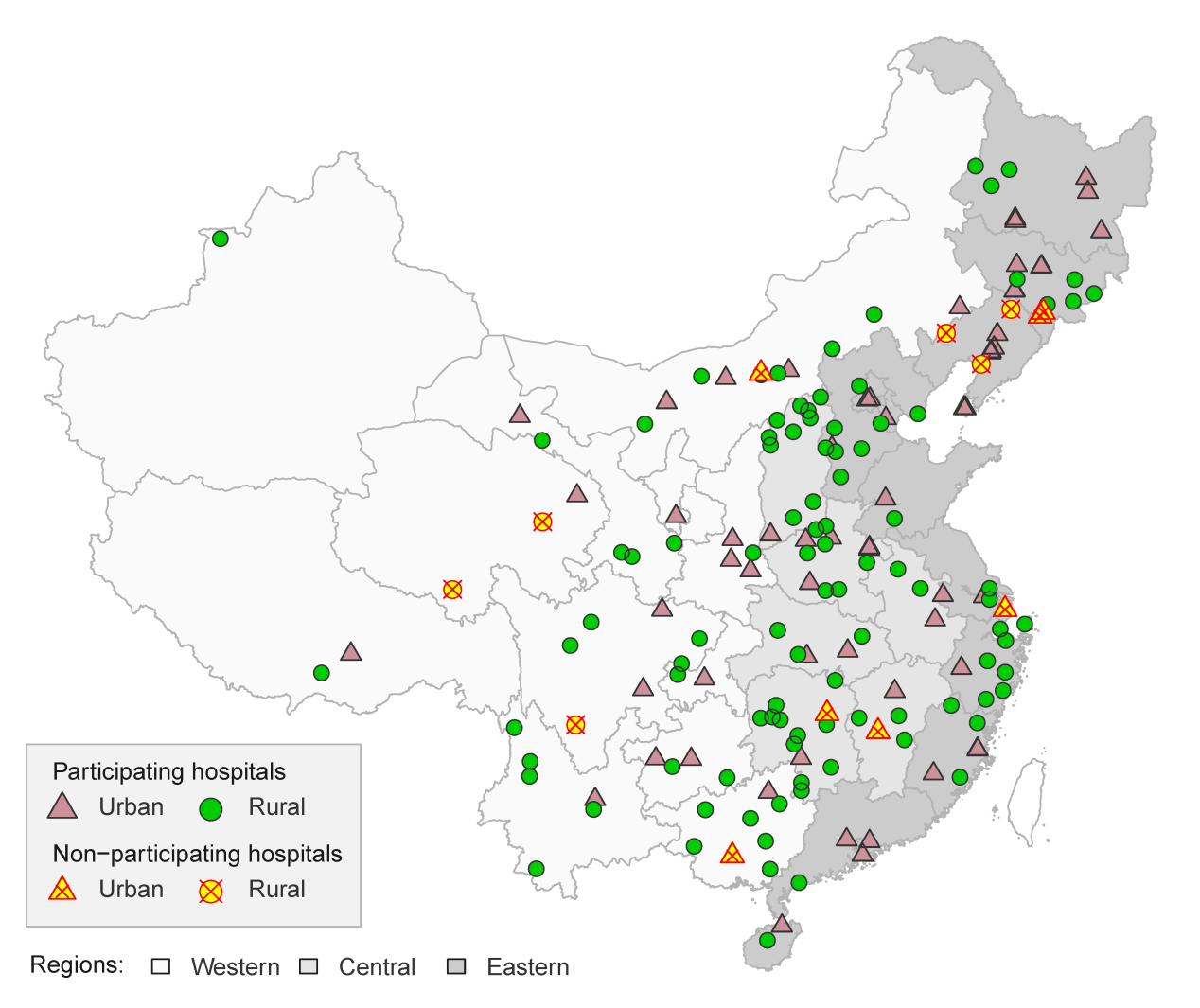


**Supplementary Fig. 1. Geographic distribution of participating hospitals in the China PEACE-Retrospective AMI study.**

**
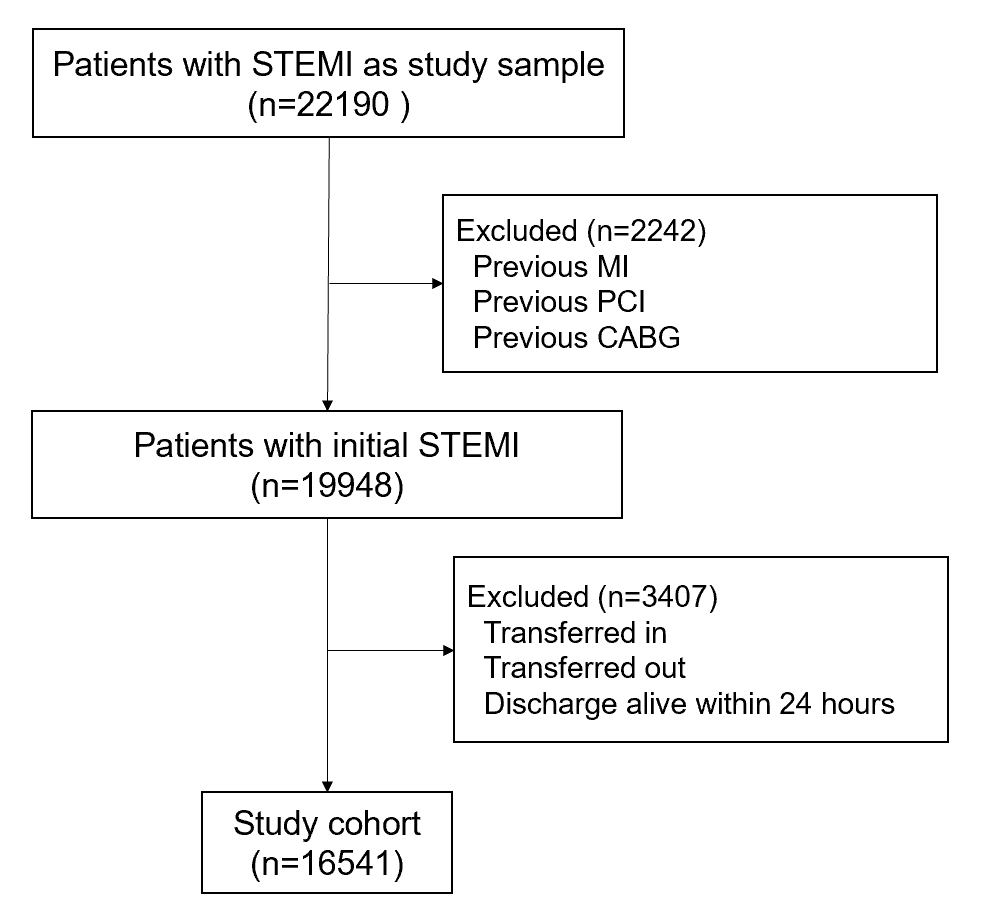
**

**Supplementary Fig. 2. Study flow chart.** STEMI indicates ST-segment elevation myocardial infarction; MI, myocardial infarction; PCI, percutaneous coronary intervention; CABG, coronary artery bypass grafting.

**Supplementary Method 1. Eligible candidates for treatments.**

Patients whose lengths of hospital stay did not exceed 24 hours were excluded for all the following treatments.

For the reperfusion therapy, we included patients who were admitted within 12 hours of symptom onset and did not receive reperfusion therapy before hospital presentation. Then we excluded patients with any contraindications (history of hemorrhagic stroke, active bleeding at presentation, or any other physician documented contraindications for fibrinolytic therapy if the patient was treated in a non-percutaneous coronary intervention [PCI] capable hospital; allergy to contrast agents or any other documented contraindication to PCI if the patient was treated in a PCI-capable hospital).

For aspirin, we excluded patients with any contraindications for aspirin (allergy to aspirin, active bleeding on admission, history of hemorrhagic stroke, or other documented contraindications).

For P2Y_12_ inhibitor, we excluded patients who participated in the ClOpidogrel and Metoprolol in Myocardial Infarction Trial (COMMIT) or patients with any contraindications for P2Y_12_ inhibitor (allergy to P2Y_12_ inhibitor, active bleeding on admission, history of hemorrhagic stroke, or other documented contraindications).

For β-blockers, we excluded patients who participated in the ClOpidogrel and Metoprolol in Myocardial Infarction Trial (COMMIT) or patients with any contraindications for β-blockers (allergy to β-blocker, cardiogenic shock on admission, heart failure on admission, second or third-degree atrioventricular block with no pacemaker implanted, systolic blood pressure <100 mmHg on admission, bradycardia [heart rate <60 beats/min] on admission without taking a β-blocker, or other documented contraindications).

For angiotensin-converting enzyme (ACE) inhibitors or angiotensin receptor blockers (ARB), we excluded patients with any contraindications for ACE inhibitors (allergy to ACE inhibitors, hyperkalemia [serum potassium>5.5 mmol/L during hospitalization], creatinine >265 umol/L during hospitalization, pregnancy or breast feeding, hypotension or other documented contraindications).

For statins, we excluded patients who were allergic to statins.

Supplementary Table 1. Proportion of patients eligible for treatment according to SMuRF status among patients who survived the first 24 hours of admission.

| **Variables**, N (%) | **SMuRF-less** | **≥1 SMuRF** | ***P* value** |
| --- | --- | --- | --- |
| In-hospital aspirin | 2929 (99.4) | 12637 (99.4) | 0.935 |
| In-hospital P2Y_12_ inhibitor | 2922 (99.2) | 12611 (99.2) | 0.802 |
| In-hospital DAPT | 2917 (99.0) | 12578 (98.9) | 0.833 |
| In-hospital β-blocker | 2197 (74.6) | 10210 (80.3) | <0.001 |
| In-hospital ACE inhibitor/ARB | 2756 (93.5) | 12228 (96.2) | <0.001 |
| In-hospital statin | 2947 (100) | 12712 (100) | 1.000 |
| In-hospital cardiac catheterization | 1627 (55.2) | 9244 (72.7) | <0.001 |
| Reperfusion therapy | 1518 (51.5) | 6925 (54.5) | 0.004 |

Abbreviations: DAPT, dual antiplatelet therapy; ACE, angiotensin-converting enzyme; ARB, angiotensin receptor blocker.

Supplementary Table 2. In-hospital outcomes according to SMuRF status in the overall population.

| **In-hospital outcomes, N (%)** | **SMuRF-less** | **≥1 SMuRF** | ***P* value** |
| --- | --- | --- | --- |
| Mortality | 608/3288 (18.5) | 1389/13253 (10.5) | <0.001 |
| Mortality within 24h | 341/3288 (10.4) | 540/13253 (4.1) | <0.001 |
| Mortality after 24h^a^ | 267/2947 (9.1) | 849/12713 (6.7) | <0.001 |
| 7-day mortality | 550/3288 (16.7) | 1173/13253 (8.9) | <0.001 |
| Composite complications^b^ | 854/3288 (26.0) | 2512/13253 (19.0) | <0.001 |
| Ventricular tachycardia/fibrillation | 156/3288 (4.7) | 563/13253 (4.2) | 0.211 |
| Major bleeding | 24/3288 (0.7) | 109/13253 (0.8) | 0.595 |
| Length of stay (days), Median (IQR) | 9 (5, 14) | 11 (7, 15) | <0.001 |

^a^ Among patients who survived after 24 hours of admission.

^b^ Composite complications include mortality, re-infarction, cardiogenic shock, ischemic stroke, or congestive heart failure.

Supplementary Table 3. Subgroup analysis for crude in-hospital mortality in the overall population.

| **Subgroup**, N (%) | **SMuRF-less** | **≥1 SMuRF** | ***P* value** |
| --- | --- | --- | --- |
| **Age** (years) |  |  |  |
| <40 | 9/79 (11.4) | 14/372 (3.8) | 0.012 |
| 40-59 | 87/789 (11.0) | 171/4345 (3.9) | <0.001 |
| 60-79 | 344/1873 (18.4) | 881/7158 (12.3) | <0.001 |
| ≥80 | 168/547 (30.7) | 323/1378 (23.4) | 0.001 |
| **Sex** |  |  |  |
| Female | 276/1104 (25.0) | 628/3866 (16.2) | <0.001 |
| Male | 332/2184 (15.2) | 761/9387 (8.1) | <0.001 |
| **Systolic blood pressure** (mmHg) |  |  |  |
| <90 | 156/317 (49.2) | 224/581 (38.6) | 0.002 |
| 90-139 | 378/2365 (16.0) | 786/7652 (10.3) | <0.001 |
| ≥140 | 66/598 (11.0) | 365/4997 (7.3) | 0.001 |
| **Cardiogenic shock at admission** |  |  |  |
| No | 439/2978 (14.7) | 1073/12450 (8.6) | <0.001 |
| Yes | 169/310 (54.5) | 316/803 (39.4) | <0.001 |
| **Chest discomfort at admission** |  |  |  |
| No | 109/348 (31.3) | 229/988 (23.2) | 0.003 |
| Yes | 499/2940 (17.0) | 1160/12265 (9.5) | <0.001 |


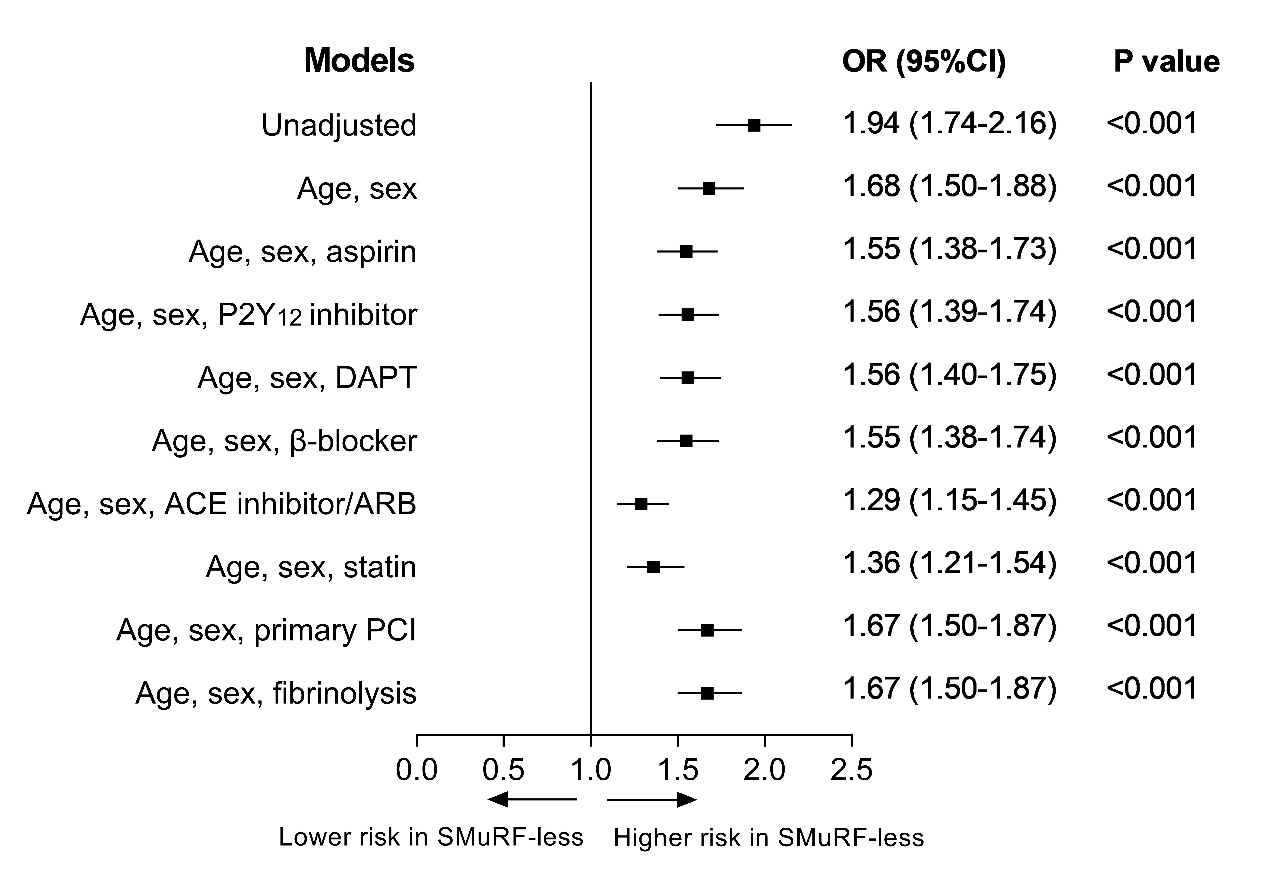


**Supplementary Fig. 3. Associations between SMuRF-less status and in-hospital mortality analyzed with mixed models in order to examine the potential effect of each treatment during hospitalization.** DAPT indicates dual antiplatelet therapy; ACE, angiotensin-converting enzyme; ARB, angiotensin receptor blocker; PCI, percutaneous coronary intervention.

**
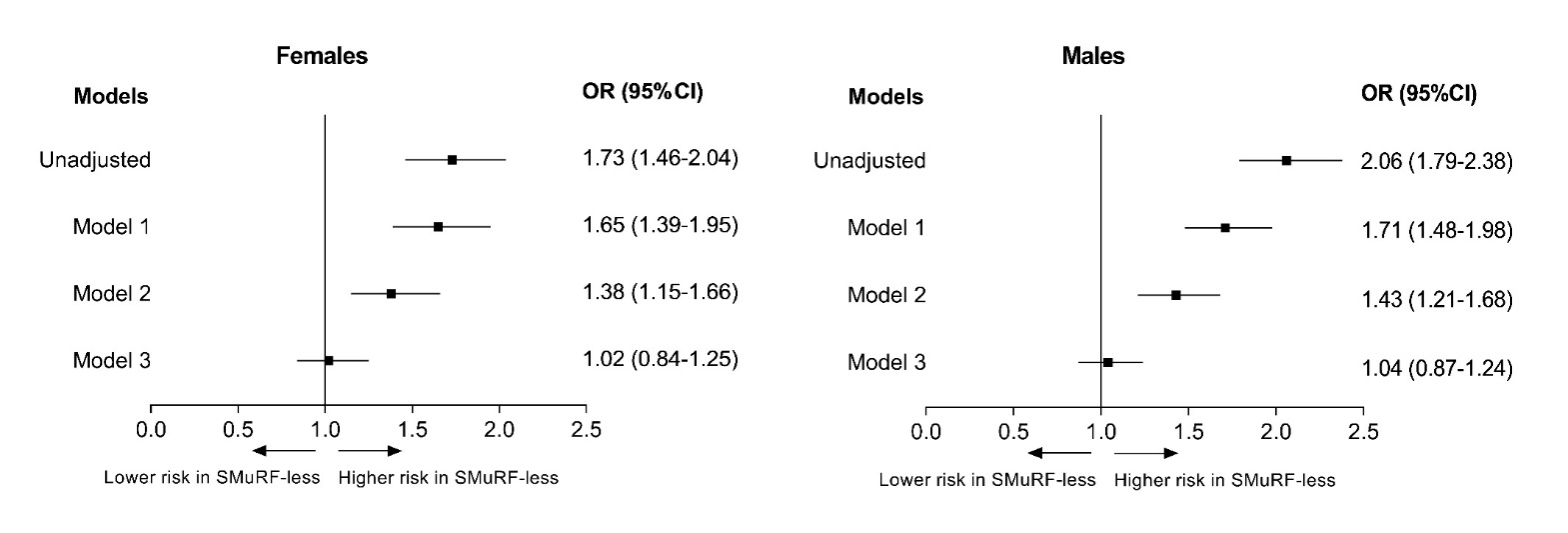
**

**Supplementary Fig. 4. Associations between SMuRF-less status and in-hospital mortality stratified by sex.** Model 1: adjusted for age; Model 2: adjusted for variables in model 1 plus clinical profiles (including previous stroke, previous atrial fibrillation, previous chronic renal disease, previous heart failure, previous peripheral arterial disease, chest discomfort, cardiac arrest at admission, cardiogenic shock at admission, stroke at admission, heart rate, and systolic blood pressure); Model 3: adjusted for variables in model 2 plus in-hospital pharmacotherapies (including aspirin, P2Y12 inhibitor, dual antiplatelet therapy, β-blocker, angiotensin-converting enzyme inhibitor/angiotensin receptor blocker, statin), and reperfusion therapy (fibrinolytic therapy, primary percutaneous coronary intervention).


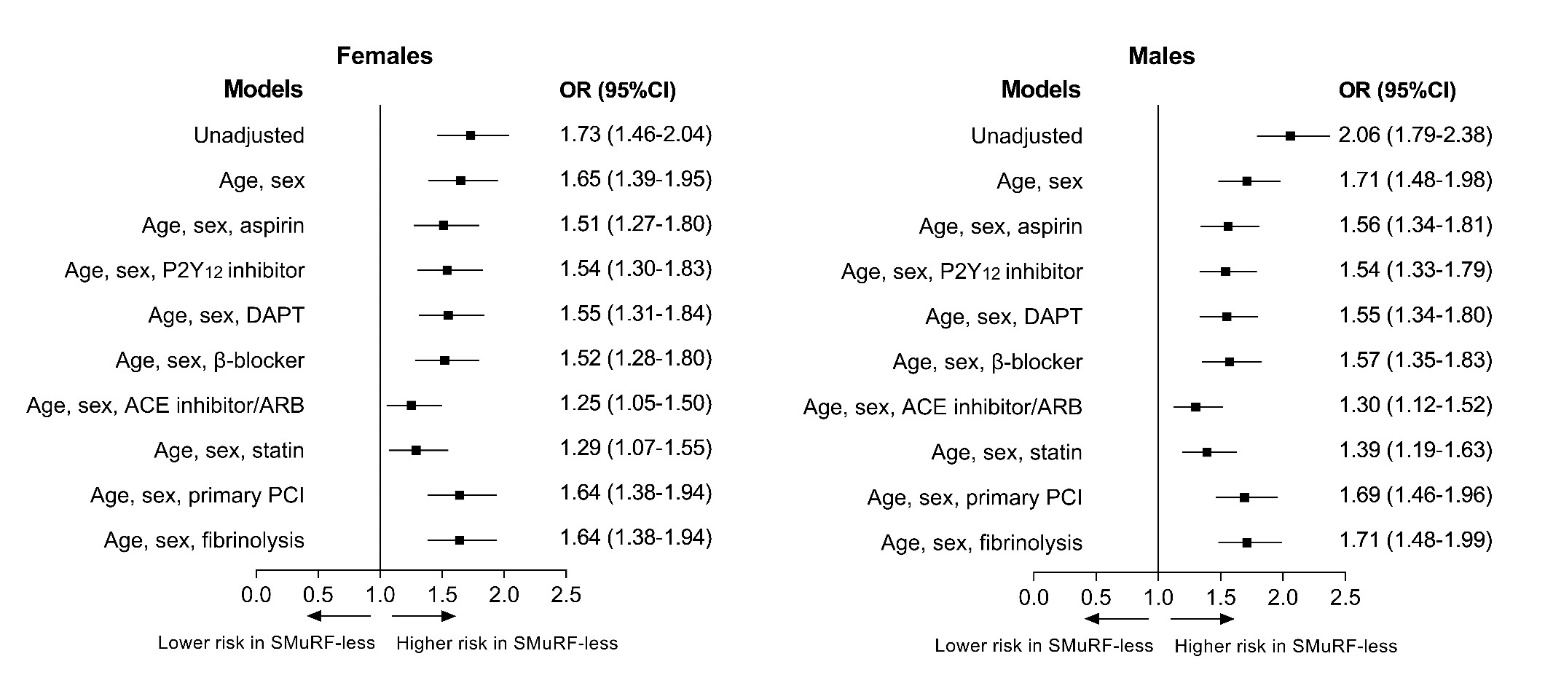


**Supplementary Fig. 5. Associations between SMuRF-less status and in-hospital mortality stratified by sex analyzed with mixed models in order to examine the potential effect of each treatment during hospitalization.** DAPT indicates dual antiplatelet therapy; ACE, angiotensin-converting enzyme; ARB, angiotensin receptor blocker; PCI, percutaneous coronary intervention.


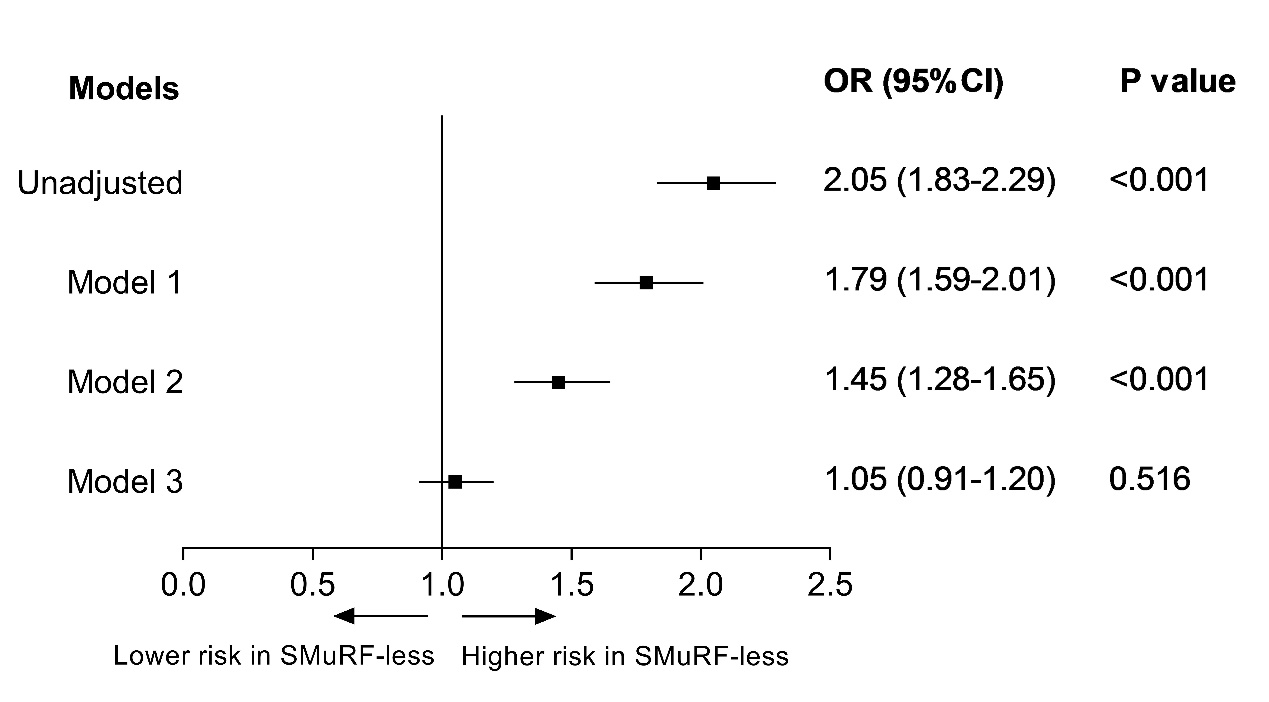


**Supplementary Fig. 6. Associations between SMuRF-less status and 7-day mortality.** Model 1: adjusted for age and sex; Model 2: adjusted for variables in model 1 plus clinical profiles (including previous stroke, previous atrial fibrillation, previous chronic renal disease, previous heart failure, previous peripheral arterial disease, chest discomfort, cardiac arrest at admission, cardiogenic shock at admission, stroke at admission, heart rate, and systolic blood pressure); Model 3: adjusted for variables in model 2 plus in-hospital pharmacotherapies (including aspirin, P2Y12 inhibitor, dual antiplatelet therapy, β-blocker, angiotensin-converting enzyme inhibitor/angiotensin receptor blocker, statin), and reperfusion therapy (fibrinolytic therapy, primary percutaneous coronary intervention).

**
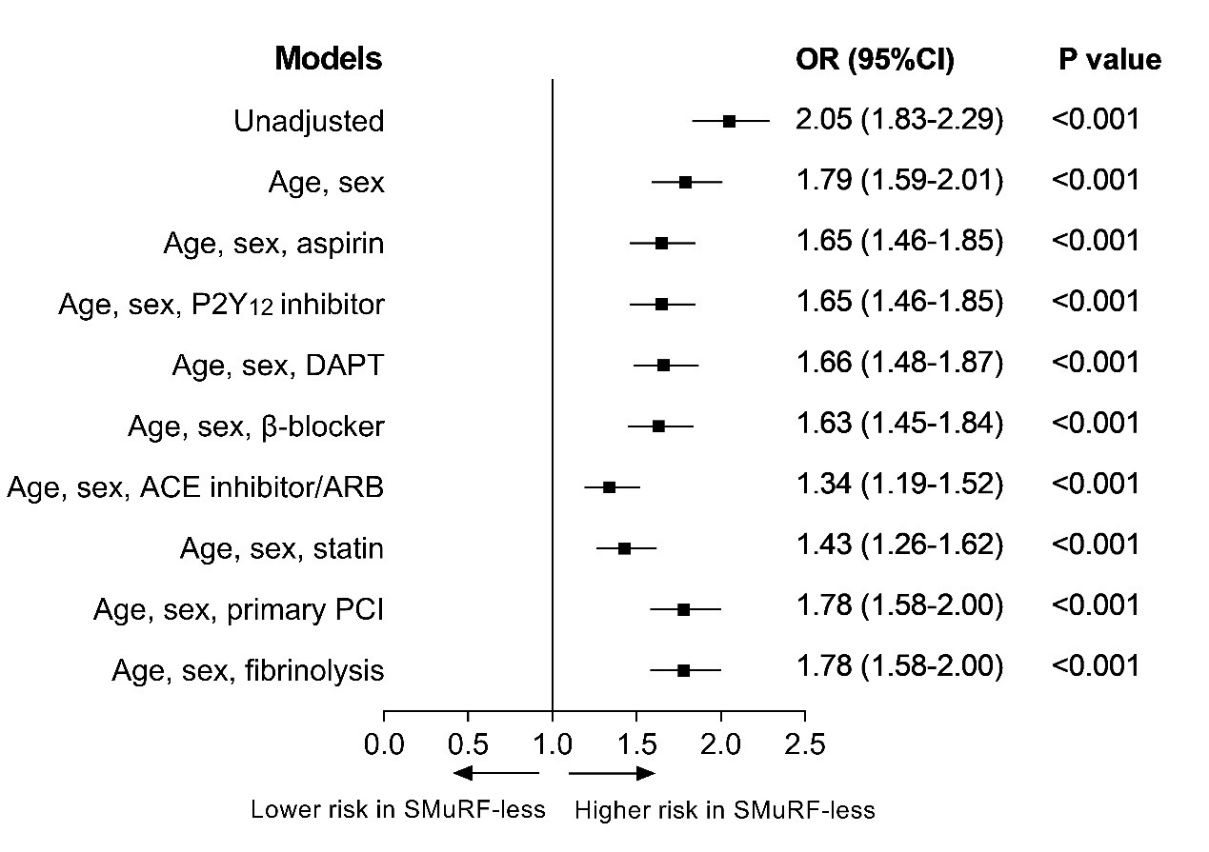
**

**Supplementary Fig. 7. Associations between SMuRF-less status and 7-day mortality analyzed with mixed models in order to examine the potential effect of each treatment during hospitalization.** DAPT indicates dual antiplatelet therapy; ACE, angiotensin-converting enzyme; ARB, angiotensin receptor blocker; PCI, percutaneous coronary intervention.


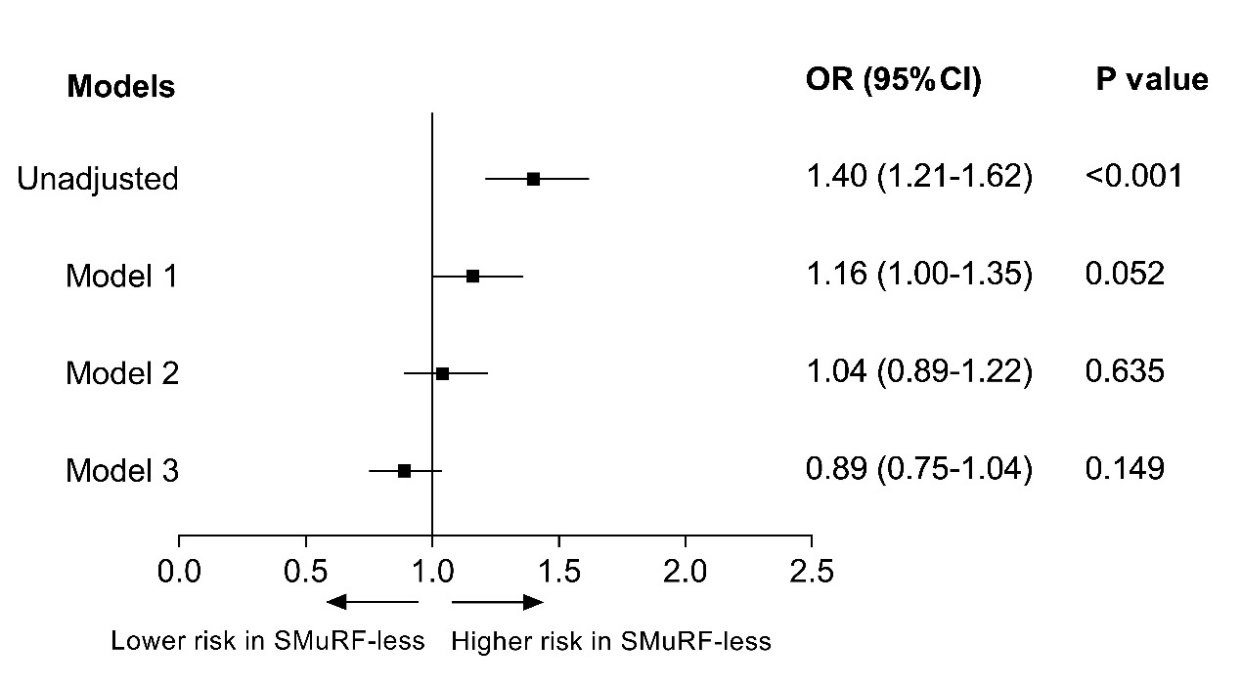


**Supplementary Fig. 8. Associations between SMuRF-less status and in-hospital mortality for patients who survived the first 24 hours of admission.** Model 1: adjusted for age and sex; Model 2: adjusted for variables in model 1 plus clinical profiles (including previous stroke, previous atrial fibrillation, previous chronic renal disease, previous heart failure, previous peripheral arterial disease, chest discomfort, cardiac arrest at admission, cardiogenic shock at admission, stroke at admission, heart rate, and systolic blood pressure); Model 3: adjusted for variables in model 2 plus in-hospital pharmacotherapies (including aspirin, P2Y12 inhibitor, dual antiplatelet therapy, β-blocker, angiotensin-converting enzyme inhibitor/angiotensin receptor blocker, statin), and reperfusion therapy (fibrinolytic therapy, primary percutaneous coronary intervention).

Supplementary Table 4. Mediation analysis of the associations between SMuRF-less status and mortality within 24 hours of admission.

|  | **Proportion mediated effect, %**^a^ |
| --- | --- |
| Total indirect effect^b^ | 57.9% |
| Indirect effect through: |  |
| Clinical profiles | 18.2% |
| Systolic blood pressure | 7.6% |
| Cardiogenic shock at admission | 1.3% |
| Treatments within 24 hours | 39.6% |
| P2Y_12_ inhibitor within 24 hours | 13.0% |
| DAPT within 24 hours | 11.9% |
| Aspirin within 24 hours | 8.5% |
| ACE inhibitor/ARB within 24 hours | 8.1% |
| Statin within 24 hours | 4.3% |
| β-blocker within 24 hours | 4.1% |
| Primary PCI | 2.5% |

^a^ The percent explained is listed for the total indirect effects of SMuRF-less status on the mortality within 24 hours of admission as compared with SMuRF status and for each mediator included in the analysis.

^b^ The sum of the percent explained effects of the individual mediators may not equal the total indirect effect because of correlation and overlapping mediation effects among mediators that is reflected in the total indirect effect but not the individual mediators.

Abbreviations: DAPT, dual antiplatelet therapy; ACE, angiotensin-converting enzyme; ARB, angiotensin receptor blocker; PCI, percutaneous coronary intervention.
